# Treatment of myelin oligodendrocyte glycoprotein antibody-associated disease (MOGAD): acute attacks, relapse prevention, and symptomatic care

**DOI:** 10.3389/fneur.2026.1965135

**Published:** 2026-10-09

**Authors:** João Vitor Mahler, Marina Vilardo, Guilherme Diogo Silva, Anastasia Vishnevetsky, Philippe-Antoine Bilodeau, Michael Levy, Marcelo Matiello

**Affiliations:** 1 Division of Neuroimmunology, Massachusetts General Hospital/Mass General Brigham, Harvard Medical School, Boston, MA, United States; 2 Division of Neuroimmunology, Department of Neurology Universidade de São Paulo, São Paulo, Brazil

**Keywords:** MOGAD, neuroimmunology, symptoms, treatment, MOG-IgG, Interleukin-6, IVIg, Plasmapheresis

## Abstract

Myelin oligodendrocyte glycoprotein antibody-associated disease (MOGAD) is a distinct inflammatory demyelinating disorder of the central nervous system, and its diagnosis is guided by the 2023 International MOGAD Panel criteria. Its clinical spectrum includes optic neuritis, transverse myelitis, acute disseminated encephalomyelitis, and brainstem or cortical encephalitis, with a phenotype distribution that is strongly age-dependent. Although attacks are typically steroid-responsive and recovery is often good, roughly half of adults relapse, and it is the accumulation of incompletely recovered attacks, rather than a progressive phase, that drives long-term disability. Management therefore has three aims: to abort and reverse the acute attack, to prevent relapses in patients at risk, and to relieve residual symptomatic disability. This review synthesizes current evidence for acute treatment (high-dose corticosteroids, therapeutic plasma exchange, and intravenous immunoglobulin), the corticosteroid taper, and maintenance immunotherapy, with attention to when treatment should be started and when it may reasonably be stopped. We summarize comparative data favoring maintenance intravenous immunoglobulin, the emerging evidence for interleukin-6 receptor blockade, the more variable performance of B-cell depletion relative to AQP4-IgG-seropositive neuromyelitis optica spectrum disorder, and the first phase 3 trials in the field (satralizumab and rozanolixizumab). We give particular attention to children, in whom acute disseminated encephalomyelitis predominates and in whom cognitive, academic, growth and skeletal outcomes must shape treatment decisions, and to the real-world availability of antibody testing and therapy outside high-income settings. Finally, we address symptomatic care: visual rehabilitation, neuropathic and spasticity-related pain, fatigue, bladder dysfunction, cognition, and mood.

## 1 Introduction

MOGAD is an antibody-associated demyelinating disease defined by serum immunoglobulin G (IgG) against conformational myelin oligodendrocyte glycoprotein, detected by cell-based assay in a patient with a compatible clinical syndrome and after exclusion of alternative diagnoses. The 2023 International MOGAD Panel criteria unified the clinical criteria around a positive MOG-IgG test, one of six core demyelinating events, and supporting clinical or MRI features, and they explicitly incorporate antibody titer because low-positive results carry a substantial false-positive rate (1).

The prevalence of MOGAD appears to be rare: population-based studies estimate a prevalence of roughly 1.3–2.5 per 100,000 and an annual incidence of about 3.4–4.8 per million, with no strong sex predominance and approximately one third of cases occurring in children. The median age at adult onset is in the early thirties, and phenotype is age-dependent, with acute disseminated encephalomyelitis (ADEM) predominating in young children and optic neuritis and myelitis in adults (2, 3).

Two features shape the therapeutic approach and distinguish MOGAD from both multiple sclerosis (MS) and AQP4-IgG-seropositive neuromyelitis optica spectrum disorder (NMOSD). First, attacks are usually exquisitely corticosteroid-responsive and often recover well, so a proportion of patients, particularly those with a monophasic course, may not require prolonged immunosuppression. Second, disability in MOGAD is almost entirely relapse-driven; progression independent of relapse activity appears extremely rare, in contrast to MS (4). The central clinical problem is therefore to identify who will relapse, to prevent those relapses, and to do so safely and without exposing selected patients to unnecessary immunotherapy.

Until recently, no therapy had been prospectively studied in MOGAD, and practice was extrapolated from retrospective cohorts and from adjacent diseases. That extrapolation has come from NMOSD but, in our experience, at least as often from MS, with which most non-specialist neurologists are far more familiar. The implicit reasoning is that an agent such as rituximab, which performs well across neuroimmunological disease in general, will perform equally well in MOGAD. This is a recurring source of suboptimal treatment selection, and one of the reasons the comparative-effectiveness data reviewed below matter clinically. This landscape is now changing with the results of two phase 3 randomized clinical trials (5, 6).

## 2 Treatment of the acute attack

### 2.1 High-dose corticosteroids and the value of early treatment

Intravenous methylprednisolone (typically 1 g daily for 3–5 days in adults, or 20–30 mg/kg/day up to 1 g in children) is the first-line treatment for an acute MOGAD attack and produces rapid, often dramatic, improvement. Because MOGAD attacks, especially optic neuritis, can be severe and because antibody results are frequently unavailable at presentation, initial treatment and escalation (when necessary) should not be delayed while awaiting serology. As AQP4-IgG-seropositive NMOSD is also possible in many of the initial presentations, testing for both antibodies at the same time is indicated (5, 6).

Timing appears to matter well beyond the speed of acute recovery. In a retrospective, nationwide, multicenter cohort of 240 adults with MOGAD treated at 14 South Korean hospitals between 2009 and 2023, the interval from first-attack onset to acute treatment was stratified as early (<5 days), intermediate (5–14 days), or late (>14 days), and shorter intervals were associated with a lower risk of a subsequent relapsing course. Compared with early treatment, late treatment carried roughly a 2.6-fold higher adjusted relapse hazard and intermediate treatment about a 2-fold higher hazard. Long-term non-steroidal immunosuppressant maintenance independently reduced relapse risk (aHR 0.24; 95% CI 0.14–0.42), yet the timing effect persisted and was higher in the subgroup that never received maintenance therapy (late vs. early aHR 3.51, 95% CI 1.64–7.50; intermediate vs. early aHR 2.68, 95% CI 1.23–5.85), arguing that the benefit of early treatment is not simply a proxy for downstream immunosuppression. Early treatment was also associated with markedly higher odds of MOG-IgG seroconversion to negative (adjusted odds ratio 7.04; 95% CI 1.58–31.41), whereas maintenance therapy was not. These findings, albeit limited by their retrospective design, adult-only and single-country cohort, and wide confidence intervals for the serological outcome, suggest that prompt, adequate acute therapy may help modify the disease trajectory (7).

### 2.2 Plasma exchange and intravenous immunoglobulin for severe or refractory attacks

When an attack is severe, vision- or cord-threatening, or responds incompletely to steroids, escalation is warranted. Therapeutic plasma exchange (TPE) or intravenous immunoglobulin (IVIG, usually 2 g/kg divided over 2–5 days) should be used (8–10). Retrospective experience and mechanistic reasoning for rapid removal (TPE) or neutralization of pathogenic IgG (IVIG) support their use, although randomized acute-treatment data are lacking.

Most protocols deliver five to seven exchanges on alternate days, but this figure should be regarded as a common practice pattern rather than a therapeutic ceiling. In the largest dedicated pediatric series, comprising 65 children treated with TPE for 78 steroid-refractory CNS demyelinating attacks, the number of procedures was determined by clinical response, and moderate-to-marked improvement was seen in 72% of children at the end of TPE and in 88.5% at 6 months; adverse events occurred in 31 of 524 procedures (5.9%) (11). The number of exchanges should therefore be individualized against the trajectory of recovery, vascular access, and hemodynamic tolerability, with additional cycles considered in patients who are still improving at the end of a standard course and early discontinuation in those who are not tolerating the procedure.

Because the therapeutic window appears to matter, escalation should not be deferred in a patient who is failing to improve over the first 3–5 days of intravenous steroids (5, 6). A systematic review and meta-analysis of 10 observational studies (1,045 patients, 1,368 attacks) found that TPE was associated with a pooled improvement of 1.43 logMAR in visual acuity (95% CI 0.63–2.24) in MOGAD optic neuritis and a clinically significant recovery rate of 94% (95% CI 88–100%) across attacks involving optic neuritis, myelitis, or cerebral involvement, with procedure-related adverse events in only 3% (95% CI 1–14%). The benefit was most consistent in steroid-refractory attacks, where the pooled improvement was 1.22 logMAR (95% CI 0.92–1.52) with no residual heterogeneity, although confounding by indication and a low to very low certainty of evidence mean these estimates remain hypothesis-generating (12). Future perspectives in the treatment of refractory attacks without response after second-line therapies with IVIG or TPE include the use of tocilizumab as a rescue option, especially in children with fulminant anti-MOG-associated encephalitis (13).

## 3 The corticosteroid taper: an under-appreciated determinant of relapse

A recurring lesson from MOGAD cohorts is that early relapses cluster around corticosteroid withdrawal. Patients are frequently corticosteroid-dependent, and relapses tend to occur when oral prednisone is tapered below approximately 10 mg/day or within the first two to 3 months after an attack. Consequently, the taper is not an afterthought but part of attack treatment itself (14, 15).

The most granular guidance to date comes from the Australasian MOGAD Study Group’s retrospective multicenter cohort of 109 children and adults diagnosed according to the 2023 International MOGAD Panel criteria (57% female, 38% pediatric; median age at onset 26 years; median follow-up 6.2 years), in which 70% relapsed at a median of 13.7 months. Using Cox models that treated the corticosteroid course as a time-varying covariate, the investigators found a clear dose–response relationship: each 1 mg/day increment in oral prednisone was associated with a 3.7% reduction in relapse hazard (95% CI 0.8–6.6%; *p* = 0.014). Sustained exposure at or above a threshold of 12.5 mg/day was associated with a 79% lower relapse hazard (HR 0.21; 95% CI 0.07–0.60), and maintaining that threshold for at least 3 months was associated with an 88% reduction compared with patients never treated in this range (HR 0.12; 95% CI 0.03–0.44; *p* = 0.0012). Reassuringly, no patient given this regimen at onset experienced a grade >3 adverse event, and the cumulative corticosteroid burden of the three-month strategy remained modest, supporting a favorable risk–benefit balance. Disease course tracked with exposure in the same direction: 13/16 (81%) of patients who spent at least 3 months above the 12.5 mg/day threshold remained monophasic at last follow-up, compared with 12/49 (25%) treated above threshold for a shorter period and 8/44 (18%) never treated in this range (*p* < 0.001). A practical default is therefore to maintain roughly 12.5 mg/day (0.16 mg/kg/day in children) for at least 3 months after onset before a slow taper. These estimates derive from a retrospective, mixed pediatric–adult cohort with wide confidence intervals in some strata, so they inform rather than mandate practice; nonetheless, they provide the first concrete, evidence-based onset-dosing recommendation in MOGAD (14).

An independent single-center cohort, likewise applying the 2023 criteria, has since reproduced this signal. Among 72 patients (41.7% pediatric) contributing 114 risk intervals, an Andersen–Gill model accounting for recurrent events found that corticosteroid exposure at ≥12.5 mg/day prednisone equivalent sustained for ≥12 weeks was independently associated with a lower relapse hazard (HR 0.30; 95% CI 0.10–0.89; *p* = 0.03), as was steroid-sparing maintenance immunotherapy (HR 0.06; 95% CI 0.02–0.23; *p* < 0.001). Corticosteroid-related adverse effects were documented in 13.5% of treated intervals and prompted discontinuation in 3.6%, concentrated among adults aged 45 years or older (16).

These data justify a deliberate rather than a rapid taper, but the risk–benefit calculation is not identical across the age range, and the favorable adverse-event profile reported in adult-weighted cohorts should not be transposed uncritically to children. In a systematic review of 101 studies of long-course oral corticosteroids in children (defined as ≥15 days of treatment), the most frequent adverse reactions were weight gain (21.1%), growth retardation (18.1%) and Cushingoid features (19.4%); reduced bone mineral density was documented in 21.5% of those in whom it was measured, biochemical hypothalamic–pituitary–adrenal axis suppression was common, and infection was the most serious adverse effect (17). Because a three-month course at 0.16 mg/kg/day falls squarely within this exposure range, children treated with a prolonged taper warrant explicit monitoring of height velocity and weight against growth charts, blood pressure and glucose, pubertal staging where age-appropriate, and bone health (calcium and vitamin D repletion, with bone densitometry in those receiving repeated or extended courses). Where relapse risk is high in a child, this is an argument for earlier introduction of a steroid-sparing agent so that the corticosteroid taper can be completed on schedule, rather than an argument against adequate initial corticosteroid exposure.

## 4 Relapse prevention: maintenance immunotherapy

### 4.1 When to start

In contrast to AQP4-IgG-seropositive NMOSD, where long-term therapy is indicated after the first attack for all patients, maintenance immunotherapy in MOGAD for every patient is a matter of debate. In some practices, maintenance immunotherapy is reserved for patients with a relapsing course, usually after a second attack, because more than half of patients, particularly children with ADEM or those with transient antibody positivity, follow a monophasic course. Exceptions are made for patients left with a severe initial attack and/or severe residual deficit after a single attack, for whom a further relapse would be poorly tolerated, and for patients with persistently high MOG-IgG titers, which are associated with a relapsing course (6, 18).

Risk stratification at onset is imperfect but improving. Early relapses predict a subsequent relapsing phenotype, and features such as persistent seropositivity, higher antibody titer, and incomplete recovery raise the index of suspicion. A reasonable stance is individualized: ‘watchful waiting’ with a written relapse action plan and early acute re-treatment for mild, fully recovered monophasic presentations, and prompt maintenance therapy for relapsing or severe disease (19). Outcome data now support the safety of that stance in selected patients: in an international incident cohort of 81 patients followed for a median of 6.3 years, 65 (80%) were observed off steroid-sparing immunotherapy after the onset attack, of whom roughly one third remained monophasic and 19% ended with an unfavorable disability outcome (EDSS ≥2.0), although most of that disability was attributable to the onset attack itself, with only 6% arising from a subsequent relapse. Nearly 40% eventually started maintenance therapy because of relapsing disease, and a third of first relapses occurred within 12 months, which argues for close surveillance and a rapid re-treatment pathway during the first year rather than for universal early immunosuppression (20).

The MOG-AR score was developed to predict the risk of relapsing disease using five domains: use of immunosuppressants, steroid duration less than 3 months, age at onset older than 45 years, female sex, and attack phenotype (lower risk for myelitis, higher risk for cortical encephalitis and optic neuritis). Scores range from 0 to 16 and are grouped into four risk grades (0–4, 5–8, 9–12 and 13–16), with a score of ≥9 proposed as the threshold identifying patients at high risk of relapse. Predicted relapse risk ranged from 33.3% (95% CI 13.2–53.5%) for scores 0–4 to 78.8% (95% CI 67.7–89.9%) for scores 13–16, with an area under the receiver operating characteristic curve of 0.709 (95% CI 0.655–0.763) in the derivation cohort (21). However, the score has two important limitations that restrict its clinical utility. First, it has not validated well outside its derivation setting: in the UK-MOGAD cohort the area under the curve was only 0.58 (95% CI 0.52–0.63) (22), and in a retrospective study across 24 Italian centers the proposed cut-off of ≥9 yielded a sensitivity of 53.9% and a specificity of 65.2% for relapse (23). Second, it is built entirely from baseline clinical variables and does not incorporate dynamic biomarkers such as MOG-IgG persistence, which is itself associated with relapse risk and which changes over time; whether adding serial serostatus would improve discrimination has not been formally tested (1, 24).

### 4.2 Choice of agent and comparative effectiveness

Because no head-to-head randomized trials exist, agent selection rests on expert opinion and retrospective cohorts, which nonetheless show a consistent hierarchy. In an early multicenter comparison of 70 patients, the proportion who continued to relapse and the annualized relapse rate (ARR) were lowest with maintenance IVIG and highest with MS disease-modifying therapies, with mycophenolate mofetil, rituximab, and azathioprine showing intermediate performance (15). MS disease-modifying drugs are ineffective in MOGAD, and several, including interferon-beta, natalizumab and alemtuzumab, have been associated with continued or increased attack activity in reported series and should not be used (25–27).

Maintenance IVIG has the strongest retrospective supporting dataset. In a large adult cohort, IVIG was associated with a significant reduction in relapses, with fewer relapses at higher or more frequent dosing; a common regimen is around 1 or 2 g/kg every 4 weeks, with the interval and dose individualized. Subcutaneous immunoglobulin is a reasonable alternative for patients who do not tolerate the intravenous infusion (28, 29).

The role of B-cell depletion is more nuanced than in NMOSD. Rituximab reduces relapse rates in many series, but its efficacy in MOGAD is consistently lower than in AQP4-IgG-seropositive NMOSD, with breakthrough relapses reported even during documented B-cell depletion, often around B-cell repopulation or at the end of the dosing interval. In a real-world cohort of patients with relapsing MOGAD, IVIG produced the lowest ARR and the highest relapse-freedom odds. Across 88 patients and more than 400 relapses, the annualized relapse rate was 0.13 (95% CI 0.06–0.27) on IVIG, 0.32 (95% CI 0.19–0.53) on mycophenolate, and 0.51 (95% CI 0.34–0.77) on B-cell depletion, with corresponding relapse-freedom probabilities of 72, 49, and 33%; restricting the analysis to IVIG dosed at 2 g/kg monthly raised relapse freedom to 85% (odds ratio 13.97 versus prednisone; 95% CI 3.09–63.17) (30). These observations have led several groups to favor IVIG first-line and to regard rituximab as a less reliable option in MOGAD than clinicians accustomed to NMOSD might expect (30).

Mycophenolate mofetil and azathioprine remain widely used steroid-sparing oral agents, particularly where IVIG or rituximab access is limited. Both have shown moderate relapse reduction in retrospective series, although neither has been tested prospectively. In the multicenter comparison above, 74% of mycophenolate-treated and 62% of azathioprine-treated patients continued to have relapses, although ARR fell in both groups (15, 25, 26). Because both take weeks to months to reach full effect, they are often combined with a low-dose oral prednisone bridge for the first three to 6 months until the agent takes effect, an approach also endorsed by regional consensus recommendations (31).

Interleukin-6 (IL-6) receptor blockade has moved from anecdote to a substantial evidence base over the past 2 years. In a pooled case series of 10 patients with highly relapsing, steroid-dependent MOGAD, most of whom had already failed rituximab, no clinical or radiographic relapses occurred over a mean of 28.6 months of intravenous or subcutaneous tocilizumab, and several patients were able to discontinue corticosteroids altogether (32). A real-world comparison of 26 patients with MOGAD found that tocilizumab lowered the annualized relapse rate (*p* = 0.008) and improved EDSS scores relative to both rituximab (*p* = 0.03) and prednisolone (*p* = 0.001), whereas rituximab did not differ significantly from prednisolone; the tocilizumab and rituximab arms comprised only eight and 11 patients respectively, so these estimates are hypothesis-generating (33).

These signals have now been substantiated by a large multicenter observational study. Across sites in the United States, Canada, Mexico, Argentina, Brazil, Chile, Colombia and Peru, 116 patients with MOGAD (89% relapsing; 18% younger than 18 years) treated with an IL-6 receptor blocker, tocilizumab in 104 (90%) and satralizumab in 12 (10%), were followed for a median of 1.4 years, during which 23 relapses occurred over 241.8 person-years. The ARR fell from 0.64 (95% CI 0.58–0.70) before treatment to 0.09 (95% CI 0.06–0.14) during treatment (incidence rate ratio 0.08; 95% CI 0.04–0.16). Using inverse probability of treatment weighting against a historical IVIG cohort (*n* = 59; ARR 0.22, 95% CI 0.15–0.32), IL-6 receptor blockade was associated with a lower relapse hazard than IVIG dosed at less than 1 g/kg every 4 weeks, but was not significantly different from IVIG at 1 g/kg or more every 4 weeks. Adverse events occurred in 50% of patients, most commonly mild infections, with severe infections in 9% (34). Two points are clinically relevant. First, the inclusion of pediatric patients and of sites across Latin America makes these data more generalizable than most MOGAD treatment series. Second, tocilizumab is widely available and comparatively inexpensive, which positions IL-6 receptor blockade as a practical option in settings where maintenance IVIG at effective doses is not affordable or not obtainable.

Surveys and consensus exercises confirm wide practice variation. An international survey of 52 MOGAD experts across 22 countries found rituximab, azathioprine, mycophenolate and IVIG all commonly chosen, with pediatric neurologists favoring IVIG more strongly than adult neurologists (35). A subsequent survey of neurologists conducted through the American Academy of Neurology found substantial variability in decision making for pediatric MOGAD after both first and second neuroinflammatory episodes, with differences between neuroimmunologists and general neurologists in both investigation and treatment (36). A Latin American RAND/UCLA modified Delphi consensus has since issued regionally adapted recommendations for adult MOGAD (31).

### 4.3 MRI monitoring

Asymptomatic lesions on MRI are rare in MOGAD (5.6% of patients), but when detected during remission are strongly associated with relapse in subsequent months (HR 11.5; 95% CI 4.0–33.0; *p* < 0.001) (37). However, the value of routine MRI monitoring, as well as the interval at which it should be repeated, remains a topic of controversy that should be better evaluated in future studies.

### 4.4 Drug monitoring and safety

For IVIG, dosing is individualized and monitoring focuses on infusion reactions, headache and aseptic meningitis, thromboembolic risk in susceptible patients, and pre-treatment screening of renal function and immunoglobulin levels. In theory, the treatment interval could be usually extended progressively, for example from four-weekly toward six- or eight-weekly, in patients who remain free of clinical and radiological activity, and shortened again if breakthrough activity occurs. Research to guide such interval change has not been conducted. In selected cases subcutaneous immunoglobulin can reduce systemic reactions and infusion burden.

For rituximab, monitoring serves three distinct purposes that are worth separating. First, CD19/CD20 counts identify B-cell repopulation, which frequently precedes late-cycle breakthrough relapses; where the assay is available, repopulation-guided rather than strictly fixed-interval re-dosing can reduce end-of-cycle attacks. Second, serum immunoglobulins should be measured before and periodically during treatment, because hypogammaglobulinemia accumulates with cumulative exposure and may itself warrant a change of agent. Third, and partly independently of immunoglobulin levels, cumulative B-cell depletion increases infection risk, so patients should be screened for hepatitis B and tuberculosis before starting and monitored for recurrent or severe infection thereafter.

Oral agents such as mycophenolate and azathioprine require frequent blood-count and liver monitoring (with thiopurine methyltransferase testing before azathioprine). Infection prophylaxis (for example for recurrent urinary tract infection) should be considered in immunosuppressed patients who are having repeated infections; such patients may also benefit from transitioning from an immunosuppressant to IVIG.

Vaccination in this population deserves more nuance than a single rule. Where the clinical situation allows, immunization should be brought up to date before immunosuppression is started, since responses to both inactivated and live-attenuated vaccines are best in the untreated state. High-dose corticosteroids (conventionally ≥20 mg/day prednisone equivalent for ≥14 days) blunt, but do not abolish, the antibody response to inactivated vaccines; this is a reason to prefer vaccination before or after a pulse and taper where feasible, and not a reason to withhold an indicated inactivated vaccine from a patient who is receiving corticosteroids. Live-attenuated vaccines should generally be avoided, especially during B-cell depletion and other immunosuppressive therapy and deferred until immune reconstitution. IVIG is an important exception to the usual live-vaccine rule: it is not immunosuppressive, so live vaccines are not contraindicated on treatment grounds, but passively transferred antibody interferes with the response to live-attenuated measles-containing and varicella vaccines, which should therefore be deferred for approximately eight to 11 months after a 2 g/kg dose. Inactivated vaccines may be given at any point relative to IVIG. Under anti-CD20 therapy, inactivated vaccines should ideally be given at least 4 weeks before the first infusion.

### 4.5 Emerging targeted therapies and the first randomized trials

MOGAD is transitioning from empiricism toward evidence. Two mechanisms dominate the current pipeline (Figure 1): interruption of IL-6 signaling, which supports plasmablast survival and antibody production, and reduction of circulating pathogenic IgG through neonatal Fc receptor (FcRn) blockade.

**Figure 1:**
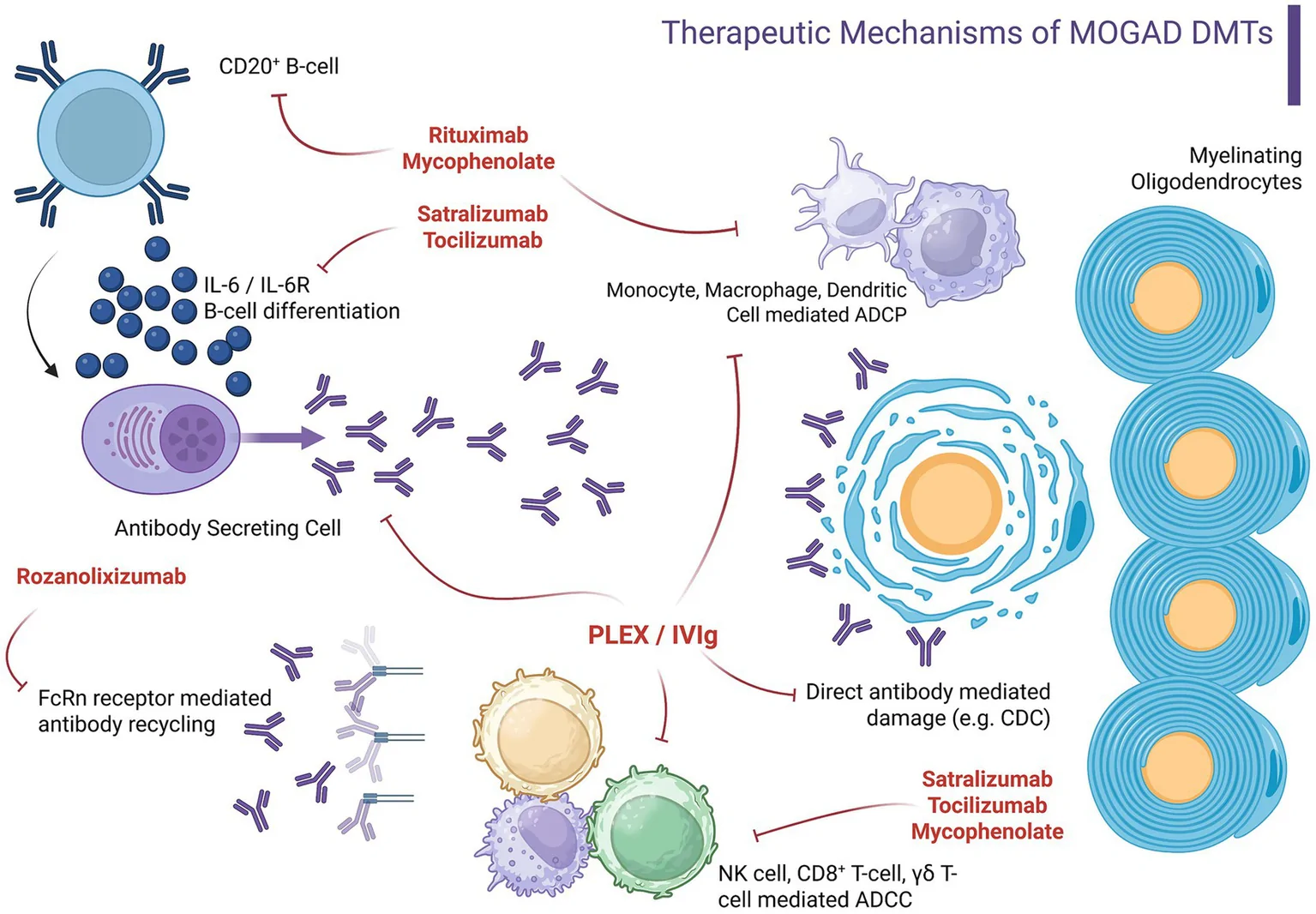
Principal points of action of established and emerging MOGAD therapies along the pathway from B-cell activation to circulating anti-MOG IgG. Naïve B cells encounter antigen and, with T-cell help, differentiate into short-lived plasmablasts and long-lived plasma cells that secrete anti-MOG IgG; secreted IgG is protected from lysosomal degradation and returned to the circulation by FcRn-mediated recycling, and effector functions are mediated through complement and Fc-receptor-bearing cells. Against this pathway: Rituximab depletes CD20 + B cells but spares CD20-negative plasmablasts and long-lived plasma cells, which is the mechanistic basis for its more variable performance in MOGAD than in AQP4-IgG-seropositive NMOSD; anti-IL-6-receptor agents (satralizumab, tocilizumab) blunt IL-6-dependent plasmablast survival, differentiation and antibody production; IVIG acts through several mechanisms, including saturation of FcRn (accelerating clearance of endogenous pathogenic IgG), neutralization by anti-idiotypic antibodies, Fc-receptor blockade and complement scavenging; therapeutic plasma exchange removes circulating IgG and complement directly and rozanolixizumab blocks FcRn-mediated IgG recycling to accelerate autoantibody catabolism. CAR T-cell and antigen-specific tolerization strategies, discussed in the text, act upstream at the level of the antibody-secreting compartment and of antigen-specific tolerance, respectively, and are not depicted. AQP4, aquaporin-4; BCMA, B-cell maturation antigen; CAR, chimeric antigen receptor; CD19/CD20, cluster of differentiation 19/20; FcRn, neonatal Fc receptor; IgG, immunoglobulin G; IL-6, interleukin-6; IL-6R, interleukin-6 receptor; IVIG, intravenous immunoglobulin; MOG, myelin oligodendrocyte glycoprotein; NMOSD, neuromyelitis optica spectrum disorder; TPE, therapeutic plasma exchange.

Satralizumab, an anti-IL-6-receptor monoclonal antibody already approved in AQP4-IgG-seropositive NMOSD, was evaluated in METEOROID, a randomized, double-blind, placebo-controlled, event-driven, multicenter phase 3 study. Participants were aged 12 years and older with relapsing MOGAD (at least one attack in the preceding 12 months or two attacks in 24 months) and were randomized 1:1 to weight-based subcutaneous satralizumab (60, 120 or 180 mg) or placebo at weeks 0, 2 and 4 and every 4 weeks thereafter, as monotherapy or added on to stable background immunosuppressive therapy, which was continued during the trial. The double-blind period was event-driven and concluded after 28 adjudicated relapses; participants who relapsed or completed the double-blind period could enter an open-label extension. The primary endpoint was time from randomization to first adjudicated MOGAD relapse. Topline results reported in 2026 indicate that the trial met its primary endpoint, with a 68% reduction in the risk of relapse relative to placebo and 87% versus 67% of participants relapse-free at 48 weeks, representing the first successful randomized controlled trial in MOGAD (38). At the time of writing, the full publication and regulatory decisions are still pending, and the estimates above should be read as preliminary.

Rozanolixizumab, a subcutaneously infused FcRn inhibitor, is under evaluation in cosMOG, a randomized, double-blind, placebo-controlled, multicenter phase 3 pivotal study with an open-label extension, enrolling adults (≥18 years) with relapsing MOGAD defined by more than one attack including one in the preceding year. In contrast to METEOROID, cosMOG is a monotherapy design: participants may not receive concomitant maintenance immunotherapy, and those who relapse on placebo may cross over to active treatment (39). The rationale is direct: any disease responsive to IVIG or plasma exchange, both of which lower pathogenic IgG, might respond to accelerated IgG catabolism through FcRn blockade. Preliminary off-label experience supports that premise in the acute setting: in a retrospective series of 27 adults with NMOSD or MOGAD treated during an attack, adding efgartigimod to intravenous methylprednisolone reduced serum IgG by 69.8% and produced a mean EDSS improvement of 1.3 points at 4 weeks compared with 0.5 points on methylprednisolone alone, with the five MOGAD patients in the efgartigimod arm improving from a mean EDSS of 4.5 to 2.7. Within the same series, an intensive schedule (20 mg/kg on days 1 and 8) produced faster EDSS improvement over the first 2 weeks than the conventional weekly 10 mg/kg regimen given in four doses, despite an identical cumulative dose, with comparable trough IgG levels in both cohorts at week 4 and no severe adverse events, although the intensive cohort also started from a lower baseline EDSS (4.5 versus 5.3), so the apparent advantage of front-loading remains uncertain. These data come from a single center, without randomization and with only 10 MOGAD patients in total, and are best read as evidence that FcRn blockade is biologically active in this disease rather than as proof of clinical benefit (40). At the time of writing, cosMOG remains ongoing, with completion now anticipated later than initially projected.

Together these trials will provide the first class-I evidence to guide attack prevention in MOGAD.

### 4.6 When to stop maintenance therapy

Because a proportion of patients become and remain relapse-free, and because immunotherapy carries cumulative infection risk and, in the case of long-term corticosteroids, metabolic and skeletal risk, de-escalation is an important and unresolved question. No randomized data define an optimal duration. In practice, withdrawal is considered after a sustained relapse-free interval, often 2 years or more, and is weighed against markers of ongoing risk (6, 41).

The French NOMADMUS study provides the most informative dataset to date. Among 83 treatment discontinuations, the cumulative risk of relapse within the first year after stopping was 8.7%, and relapses that did occur were generally mild. Two aspects of that cohort merit emphasis. First, discontinuation was scheduled, that is, planned on the basis of clinical stability, in only 65% of cases, while 35% were driven by adverse events; relapse risk did not differ significantly between these two groups. Second, the lowest risk was seen in patients who had achieved at least 2 years of clinical stability before stopping (24).

MOG-IgG serostatus informs but does not dictate the decision. Seroconversion to negative is associated with a lower relapse risk and is more frequent after early acute treatment and after some maintenance therapies, yet seronegative patients can still relapse and persistently seropositive patients can remain monophasic; antibody status is therefore a supportive rather than a definitive biomarker. A reasonable approach is to individualize and attempt cautious withdrawal in patients who are durably relapse-free and seronegative, maintain therapy in those with persistently high titers or a history of severe relapses, and ensure that any patient coming off therapy has a clear plan for rapid acute re-treatment should symptoms recur (6, 19). Importantly, this strategy presupposes access to serial cell-based-assay testing, which, as discussed in Section 6.3, cannot be assumed in many parts of the world.

## 5 Symptomatic and rehabilitative management

Attack prevention addresses only part of the disease burden. Many patients live with residual visual, sensory, motor, autonomic, cognitive and affective symptoms, and this domain is consistently under-treated. Symptomatic care should be integrated from the outset rather than deferred.

### 5.1 Residual visual impairment and low-vision rehabilitation

Optic neuritis is the commonest adult presentation, frequently bilateral and with striking disc edema, but visual prognosis in MOGAD is generally more favorable than in AQP4-IgG-seropositive NMOSD. Even when optical coherence tomography documents permanent peripapillary retinal nerve fiber layer thinning, which develops over the first three to 4 months and then stabilizes, visual acuity often recovers well, and in pediatric cohorts recovery is frequently complete. Prompt, adequate acute treatment of each episode is the single most important determinant of the visual outcome, underscoring the link between attack care and function. In 82 adults from the French MOGAD cohort with inaugural optic neuritis, 91% regained 20/20 acuity at 3 months, but failure to do so was strongly associated with a delay to first methylprednisolone infusion of ≥10 days (odds ratio 16; 95% CI 1.14–213; *p* = 0.01). The same delay was reflected structurally: peripapillary retinal nerve fiber layer thickness at 3 months averaged 75 μm in those treated within 10 days versus 66.5 μm in those treated later (*p* = 0.02), corresponding to a 15% versus 34% reduction relative to the unaffected eye (*p* < 0.001). The very wide confidence interval around the functional estimate reflects how few patients failed to recover, but the structural gradient is consistent and argues that treatment delay costs axons even when acuity ultimately returns (42).

For the minority left with fixed deficits, management should be as structured as for any other residual symptom. Refraction and contrast sensitivity should be optimized first, since uncorrected refractive error and reduced contrast compound low acuity. Formal low-vision rehabilitation has an established evidence base in other optic neuropathies and should be offered rather than assumed unnecessary because acuity is “only” moderately reduced; it includes magnification aids, high-contrast and large-print materials, screen-reader and text-to-speech software, eccentric-viewing training in those with central scotoma, and lighting and glare management. Uhthoff-type heat sensitivity is managed by avoiding rapid rises in core temperature, cooling strategies during exercise, and counselling that the phenomenon is reversible and does not signify a relapse. Occupational and educational adaptation, driving assessment where relevant, and registration for visual impairment services where eligible complete the package. Colour vision and visual field loss should be documented, as these may limit occupational function even when Snellen acuity is preserved. Unfortunately, there is no remyelinating therapy of proven benefit (5, 6).

### 5.2 Pain: neuropathic, spasticity-related, and attack-associated

Pain is common and frequently overlooked. In a dedicated study, roughly half of adults with MOGAD reported disease-related pain, either nociceptive, neuropathic, or mixed. Neuropathic pain carried the highest intensity and the greatest impairment of daily activities and physical quality of life; spasticity-associated pain and short-lasting painful tonic spasms also occurred. Pain severity, alongside visual and gait impairment, independently predicted worse physical quality of life. Importantly, pain and mood symptoms were often inadequately controlled in routine care (43).

Management follows general neuropathic-pain principles, since MOGAD-specific analgesic trials are lacking. Central neuropathic pain from myelitis is treated with agents such as gabapentinoids (gabapentin, pregabalin), tricyclic antidepressants (for example amitriptyline or nortriptyline), or serotonin–noradrenaline reuptake inhibitors (duloxetine or venlafaxine), titrated to pain control and tolerance. Painful tonic spasms characteristically respond to sodium-channel-blocking anticonvulsants such as carbamazepine or oxcarbazepine at relatively low doses. Retrobulbar or ocular-movement pain during acute optic neuritis is typically severe but short-lived and improves with corticosteroid treatment of the attack. Cognitive-behavioral therapy and a structured, stepwise analgesic plan, with attention to sleep and mood, should be offered rather than leaving pain to be managed reactively.

### 5.3 Spasticity, bladder, bowel, and sexual dysfunction

Residual myelitis produces spasticity, weakness, and gait impairment. In a UK cohort after a median disease duration of 28 months, 47% of patients were left with permanent disability: 7% had limited mobility (EDSS ≥4.0), 28% had permanent bladder issues, 20% had bowel dysfunction, and 21% of males had erectile dysfunction (44). Physiotherapy and a structured exercise program are foundational; pharmacological options for spasticity include baclofen and tizanidine, with focal botulinum toxin for troublesome regional spasticity. Neurogenic bladder with urgency, incontinence, or incomplete emptying warrants proactive assessment because it drives both quality-of-life impairment and morbidity from recurrent infection. Management ranges from antimuscarinic or beta-3 agonist agents and pelvic-floor training to intermittent catheterization, and specialist urology referral is important. Constipation and neurogenic bowel are managed with an individualized bowel regimen. Sexual dysfunction should be enquired about directly, as patients rarely raise it unprompted.

### 5.4 Fatigue, mood and cognition

More than 70% of patients with MOGAD suffer from fatigue and more than 40% from depressive symptoms, moderate to severe in one-third of patients (43, 45). In cohort data, depression was the dominant driver of reduced mental quality of life, yet often went untreated. Fatigue is managed with sleep optimization, graded activity, treatment of contributing factors (mood, pain, nocturia), and, where appropriate, a trial of pharmacological measures as used in other demyelinating diseases.

Screening for and treating depression and anxiety, both pharmacologically and with psychological support, is a core part of MOGAD care, not an optional extra. Approximately one in five adult patients with MOGAD experience notable cognitive impairment, with deficits most prevalent in mental flexibility, attention, and verbal working memory, particularly after encephalitic or cortical presentations (46). These patients may benefit from neuropsychological assessment, cognitive rehabilitation and social support. Cognitive outcomes in children are addressed separately in Section 6.1, because the assessment tools, the timescale over which deficits emerge, and the interventions required all differ.

### 5.5 Patient support, education, relapse action plans, and multidisciplinary care

Because MOGAD disability is relapse-driven and because early acute treatment improves outcomes, empowering patients to recognize and act on new symptoms is itself a therapeutic intervention. Every patient, especially those managed by watchful waiting without maintenance therapy, should leave the clinic with a written relapse action plan that describes the symptoms that warrant urgent assessment (new or worsening visual loss, limb weakness or numbness, bladder dysfunction, or intractable pain), a clear route to rapid evaluation, and the expectation that a genuine attack should be treated promptly rather than observed.

Patients also benefit from understanding the distinction between a true relapse and transient pseudo-relapse phenomena provoked by heat, infection, or fatigue, which do not require immunotherapy.

Optimal care is multidisciplinary, drawing on neurology, neuro-ophthalmology, physiotherapy and occupational therapy, urology, pain and rehabilitation medicine, and peer and mental-health support, coordinated so that symptomatic and preventive strategies are delivered together. Engagement with patient organizations and peer support groups can reduce isolation and increase access to reliable information in a rare and at times unpredictable disease.

Finally, for any patient exposed to prolonged corticosteroids, bone protection (calcium and vitamin D, bone-density monitoring, and bisphosphonates where indicated), together with attention to weight, glucose, blood pressure, and infection prophylaxis, is an essential accompaniment to immunotherapy (17, 43, 45).

## 6 Special populations and global considerations

### 6.1 Children

Children account for roughly a third of MOGAD cases, and pediatric disease differs from adult disease in ways that change management rather than merely colouring it (2, 3). ADEM is the dominant onset phenotype in young children, and the course is more often monophasic with apparently good motor and visual recovery, which is the main reason for reluctance to commit a child to indefinite immunosuppression after a single event. When maintenance therapy is required, IVIG and mycophenolate are the most frequently chosen agents, with pediatric neurologists favoring IVIG more strongly than adult neurologists (35, 36). The recent IL-6 receptor blockade cohort, in which 18% of participants were younger than 18 years, provides the first substantial pediatric safety and effectiveness signal for that class (34).

Apparent neurological recovery in a child, however, should not be read as complete recovery. The developing brain is injured during a period of active myelination and network formation, and deficits may become evident only as academic demands increase. In a single-center series of 51 children with MOGAD (median age 8 years, median follow-up 31 months, 55% presenting with ADEM), 39.5% required academic or educational intervention at last follow-up, in the form of accommodations or a learning support assistant, a significant increase over the pre-morbid state, and this was seen across phenotypes rather than only after ADEM; formal testing with the Wechsler Intelligence Scale for Children, Fifth Edition, showed overall scores below the population mean (47). Consistent with this, the European pediatric MOG consortium has reported memory impairment, attention deficits, poor concentration and learning or academic difficulties in approximately 10–50% of pediatric patients with MOGAD, a range that reflects genuine heterogeneity as well as small and methodologically varied cohorts; deficits are linked to relapses, may emerge over time, and correlate inconsistently with brain lesion burden on MRI (46, 47). Two practical implications follow. First, cognitive, executive, attentional and academic function should be assessed longitudinally rather than once, ideally at baseline after recovery from the index attack and again at transition points in schooling, with a low threshold for formal neuropsychological evaluation after ADEM, cortical encephalitis, or any relapse. Second, findings need to be translated into school-based support such as individualized education plans, extra time, reduced processing-speed demands and fatigue management within the school day, because the functional cost of MOGAD in a child is frequently educational rather than ambulatory.

Corticosteroid exposure also carries a different calculus in children, and this is particularly relevant given the evidence reviewed in Section 3 favoring a prolonged taper. Beyond the growth, weight, bone-density, metabolic and infection risks quantified above (17), clinicians should monitor pubertal development in adolescents and consider the psychological effects of Cushingoid appearance in a school-age population, which are a common reason for non-adherence. Practically, this argues for weight-based rather than fixed dosing, documentation of height velocity on growth charts at every visit, routine calcium and vitamin D repletion, and a lower threshold for introducing a steroid-sparing agent so that prolonged corticosteroid exposure is not the default relapse-prevention strategy in a growing child.

### 6.2 Pregnancy and the peripartum period

In women of childbearing age, treatment planning should account for the dynamic relapse risk across pregnancy: although relapse risk decreases significantly during gestation, it rises again in the peripartum and postpartum periods. In cohorts maintained on preventive therapies, postpartum relapse risk remains low, and IVIG represents a practical and pregnancy-compatible option (48, 49). Table 1 summarizes labelling status, placental transfer and practical use around pregnancy for the immunotherapies commonly used in MOGAD.

**Table 1:** Immunotherapies used in MOGAD and their status around pregnancy.

Agent	Labelling status	Placental transfer and fetal risk	Practical use around pregnancy in MOGAD
Corticosteroids (prednisone, methylprednisolone)	Lowest effective dose advised. Former FDA category C; TGA A (prednisolone)	Non-fluorinated corticosteroids are largely inactivated by placental 11B-HSD2, giving roughly 10% fetal exposure. Historical oral-cleft signal not confirmed in recent cohorts. Prolonged high dose: gestational diabetes, hypertension, PPROM, growth restriction	Compatible in all trimesters. IV methylprednisolone remains first-line for acute attacks; continue maintenance prednisone at the lowest effective dose. Consult the obstetric team before initiation (48)
Intravenous immunoglobulin	No fetal-risk signal; no contraindication	Actively transported across the placenta from the second trimester; no teratogenic or fetal-harm signal	Preferred maintenance option through pregnancy and postpartum; compatible with breastfeeding (48, 49)
Azathioprine	Fetal-harm language retained in labelling; no REMS. Former FDA category D; TGA D	Fetal liver lacks inosinate pyrophosphorylase, limiting conversion to active metabolites. The legacy category D reflects animal data at high dose rather than human cohort evidence	Usable despite the legacy category; compatible with lactation (48)
Mycophenolate mofetil/mycophenolic acid	Boxed warning; FDA REMS programme. Former FDA category D; TGA D	Established human teratogen: cleft lip and palate, microtia, external auditory canal atresia, micrognathia, microphthalmia, coloboma, hypertelorism, complex cardiac anomalies, oesophageal atresia, diaphragmatic hernia and skeletal anomalies. Malformation reported in 11.6 and 26% of first- trimester exposures in the two largest cohorts, with increased first- trimester loss	Contraindicated. Two negative pregnancy tests 8–10 days apart before initiation; reliable contraception during treatment and for 6 weeks after cessation. Switch to an alternative agent before conception (48)
Rituximab	No teratogenicity signal; label advises contraception during treatment and for 12 months after the last dose. Former FDA category C	IgG1: negligible first-trimester transfer, rising from about 16 weeks, with fetal levels approaching maternal by 26 weeks. Second- and third-trimester exposure can deplete neonatal B cells	Dose before conception and allow depletion to carry through pregnancy. The 12-month washout is widely regarded as over-conservative. If exposure occurs after the first trimester, monitor infant CD19 counts and defer live vaccines (48)
Tocilizumab	Data insufficient to determine risk of major birth defects or miscarriage; pregnancy exposure registry available. Former FDA category C; TGA C	Abortion and embryo- fetal death in cynomolgus monkeys during organogenesis. Actively transported across the placenta in the third trimester and may affect infant immune response	Continue while attempting conception and discontinue once pregnancy is confirmed; some guidance advises stopping 3 months (5.5 half-lives) before conception. Contraception for approximately 3 months after the last dose (48)
Satralizumab	No adequate human data; IgG2/4 anti- IL-6 receptor antibody	Placental transfer expected from the second trimester as for other IgG monoclonals; no human pregnancy dataset in MOGAD	No evidence base to guide use. Where relapse risk is high, an individualized discussion is required; pregnancy registry enrolment is advised (48)
Rozanolixizumab	No adequate human data; FcRn blockade	FcRn blockade reduces the transport that supplies fetal IgG, a theoretical concern with no human pregnancy data	Avoid pending data; plan a switch before conception (39)

The US Food and Drug Administration letter categories (A/B/C/D/X) were replaced by the narrative Pregnancy and Lactation Labelling Rule, phased out fully by June 2018; former categories are given only for orientation and should not be used for counselling. Many labels are more conservative than the accumulated clinical experience supports. Entries are derived from current product labelling together with the systematic literature review of pregnancy in MOGAD and AQP4-IgG-seropositive NMOSD and from two-center relapse-risk data across pregnancy (48, 49). 11β-HSD2, 11β-hydroxysteroid dehydrogenase type 2; CD19, cluster of differentiation 19; FcRn, neonatal Fc receptor; FDA, US Food and Drug Administration; IgG, immunoglobulin G; IL-6, interleukin-6; IV, intravenous; PPROM, preterm premature rupture of membranes; REMS, Risk Evaluation and Mitigation Strategy; TGA, Australian Therapeutic Goods Administration.

### 6.3 Global access to diagnosis and treatment

Much of the management framework set out above presupposes resources that are not universally available: dual AQP4-IgG and MOG-IgG testing at presentation, titer-informed application of the 2023 criteria, serial serostatus to guide continuation or withdrawal of therapy, and maintenance IVIG as the preferred agent. Live cell-based assay, the reference method for MOG-IgG, is concentrated in a small number of reference laboratories; in Latin America, restricted availability means that fixed cell-based assays are often used instead, that MOG-IgG is frequently tested only after a negative AQP4-IgG result rather than in parallel, and that turnaround times can exceed the window in which acute treatment decisions must be made (50, 51). Cost, geographic distance from a testing centre and sample-transport logistics compound the problem, and comparable constraints apply across many low- and middle-income settings.

The practical consequences are threefold. First, acute treatment should never be withheld pending serology, a point that applies with even greater force where results take weeks. Second, strategies that depend on serial antibody titers, in particular serostatus-guided withdrawal of maintenance therapy, may simply not be executable, and decisions in these settings will rest more heavily on clinical stability and attack severity. Third, agent selection must be realistic: where maintenance IVIG at 1–2 g/kg every 4 weeks is unaffordable or unobtainable, mycophenolate, azathioprine and, increasingly, tocilizumab are the pragmatic alternatives, and the recent multinational IL-6 receptor blockade data are directly relevant here because tocilizumab is both widely licensed and comparatively inexpensive (31, 34). Recommendations generated in high-income settings should be read with these constraints in mind, and regional consensus documents that adapt them explicitly are a valuable complement to international guidance.

## 7 Summary and future directions

MOGAD care rests on three pillars. Acute attacks should be treated early and adequately with high-dose corticosteroids, escalating to plasma exchange or IVIG when there is no improvement after a course of steroids, followed by a deliberate oral corticosteroid taper, maintaining roughly 12.5 mg/day of prednisone (0.16 mg/kg/day in children) for at least 3 months, because premature withdrawal precipitates early relapse. In children this benefit must be balanced explicitly against growth, bone, metabolic and pubertal risk.

Maintenance immunotherapy should, in most cases, be used for relapsing or severe disease and stratified by relapse risk, with current evidence favoring IVIG and IL-6 receptor blockade and cautioning that B-cell depletion is less reliable than in AQP4-IgG-seropositive NMOSD; the first randomized trials of satralizumab and rozanolixizumab are transforming this empirical landscape. Decisions to stop therapy remain individualized, informed but not dictated by serostatus, and constrained in many parts of the world by the availability of the assays on which such decisions depend.

Throughout, symptomatic and rehabilitative care, of vision, pain, spasticity, bladder, fatigue, cognition and mood, must be delivered in parallel with immunotherapy, because it is these residual symptoms that most shape patients’ daily lives. In children, the most important of these is cognitive and academic function, which requires longitudinal assessment and school-based support rather than a single post-attack evaluation.

Looking beyond the current trial landscape, the ambition is to move from broad immunosuppression toward durable, antigen-specific control, and two strategies are furthest advanced. The first is deep B-lineage depletion with chimeric antigen receptor (CAR) T cells. This is mechanistically attractive in MOGAD, where the relative underperformance of anti-CD20 therapy suggests that MOG-IgG is sustained in part by CD20-negative plasmablasts and long-lived plasma cells that rituximab spares, whereas CD19- and B-cell maturation antigen (BCMA)-directed constructs reach those compartments. Early phase 1 experience in adjacent antibody-mediated disease is encouraging: in an open-label phase 1 trial of the anti-BCMA construct CT103A in 12 patients with relapsed or refractory AQP4-IgG-seropositive NMOSD, 11 remained relapse-free over a median 5.5 months with declining serum AQP4-IgG, albeit with cytokine release syndrome in all patients and infection in 58% (52), and anti-CD19 CAR T cells have produced marked improvement in refractory generalized myasthenia gravis (53). Whether lymphodepletion, cytopenias, and infection risk can be justified in a disorder that is frequently monophasic and rarely fatal remains the central question. Transiently expressed, mRNA-engineered CAR T cells, and chimeric autoantibody receptor (CAAR) T cells that delete only MOG-specific B cells, may eventually offer a more favorable ratio.

The second strategy is antigen-specific tolerization, using myelin peptide-coupled autologous leukocytes, tolerogenic dendritic cells, or tolerogenic nanoparticles to restore regulatory rather than effector responses. A first-in-human trial in MS infused autologous peripheral blood mononuclear cells chemically coupled to seven myelin peptides, including MOG1–20 and MOG35–55, establishing feasibility and acceptable tolerability, with antigen-specific T-cell responses reduced at higher cell doses (54). Here MOGAD is arguably a more tractable target than MS, because the dominant autoantigen is known and singular, and MOG peptides already feature in the tolerization constructs tested in MS. Both approaches, however, presuppose the ability to identify in advance the minority of patients whose disease justifies them, which returns the field to the same unmet need for validated relapse-risk biomarkers.
